# Supplementary material for: A high-resolution mRNA expression time course of embryonic development in zebrafish
Source: eLife. 2017 Nov 16;6:e30860. doi: 10.7554/eLife.30860 (PMC5690287; doi:10.7554/eLife.30860)
Supplement: Supplementary file 6. [file elife-30860-supp6.zip › biolayout-clusters-files/Cluster084.html]

Cluster084


# Cluster084: Detail

### Go to ZFA detail

## GO

| GO ID | Description | Domain | Annotated | Expected | Observed | Adjusted p-value | Genes | Ensembl IDs |
| --- | --- | --- | --- | --- | --- | --- | --- | --- |
| GO:0001764 | neuron migration | biological\_process | 17 | 0.02 | 2 | 0.041 | vangl2 supt5h | ENSDARG00000027397 ENSDARG00000042724 |
